# Triplet Excitons Unlock Electroluminescence from Insulating Lanthanide Nanocrystals for Light-Emitting Diode Applications

**DOI:** 10.34133/research.1189

**Published:** 2026-03-16

**Authors:** Wenkai Li, Wei Lian, Datao Tu

**Affiliations:** ^1^ State Key Laboratory of Structural Chemistry, and Fujian Key Laboratory of Nanomaterials, Fujian Institute of Research on the Structure of Matter, Chinese Academy of Sciences, Fuzhou 350002, China.; ^2^ College of Chemistry and Materials Science, Fujian Normal University, Fuzhou 350007, China.

## Abstract

Lanthanide nanocrystals hold exceptional promise for electroluminescence applications due to their unique optical properties. However, their intrinsic insulating character and localized 4f orbitals severely restrict carrier injection, thereby hindering direct electrical excitation. In a recent study published in *Nature*, Tan and colleagues circumvented this fundamental bottleneck via molecular engineering of the nanocrystal surface. They developed a series of functionalized ligands (e.g., carbazole-phosphine oxide) to establish an electroactive interface, facilitating efficient transfer of electro-generated triplet excitons to lanthanide ions. Notably, a wide-ranging multicolor electroluminescence from lanthanide nanocrystals was achieved for the first time, exhibiting high power efficiency and external quantum efficiency. These findings provide new opportunities for electrically driven luminescence in lanthanide nanocrystals or other insulating systems.

Electroluminescence (EL), which enables conversion of electrical energy to light, plays a pivotal role in modern displays, sensing, and communication systems [1]. There is an emerging demand for EL emitters that integrate high color purity, spectral tunability, and operational stability. There is an emerging demand for electroluminescent emitters integrating high color purity, spectral tunability, and operational stability, alongside the requirement for simplified device architectures compatible with scalable manufacturing processes. Despite remarkable advancements in conventional material platforms including organic emitters, quantum dots, and perovskites, these systems still present inherent limitations in exciton manipulation and color purity, and generally necessitate complex multilayer device architectures customized for distinct emission wavelengths. These constraints impede their further advancement in the development of high-performance and scalable electroluminescent devices [2–4].

In sharp contrast, lanthanide (Ln^3+^)-based EL inherently overcomes these bottlenecks via its unique material and mechanistic attributes. Originating from parity-forbidden, shielded 4f–4f electronic transitions, Ln^3+^ emitters exhibit ultra-narrowband emission with ultrahigh color purity, as well as exceptional photothermal and chemical stability [5,6]. Nevertheless, the realization of efficient EL from Ln^3+^-doped nanocrystals has been fundamentally impeded by their intrinsic electrical insulation. The prevalent fluoride host lattices (e.g., NaLnF_4_) are wide-bandgap (~8 eV) insulators, which severely obstruct charge transport. Additionally, the localized 4f orbitals significantly impede direct carrier injection, as their electronic transitions are spin-forbidden and poorly coupled to the conduction band. These intrinsic limitations have hindered the development of Ln^3+^-based EL devices.

Recently, Tan and coworkers have demonstrated efficient EL from insulating Ln^3+^-doped fluoride nanocrystals (NaGdF_4_:X^3+^; X = Tb, Eu, Nd) by modifying them with carbazole–phosphine oxide (ArPPOA) ligands. These strategically designed ligands facilitate fast and directional energy transfer to localized 4f levels of Ln^3+^ ions, thus electrically triggering Ln^3+^ ions emission without the need for conventional carrier-injection layers [7]. The authors exquisitely designed ArPPOA ligands with different substitution groups: H(TPPOA), carbazole (CzPPOA), 3,6-di(tert-butyl)carbazole (tBCzPPOA), 9,9-dimethylacridine (DMACPPOA), and 9,9-diphenylacridine (DPACPPOA), which contain electron-donating groups to adjust the energy levels of frontier molecular orbitals to match the energy levels of Ln^3+^ ions for efficient EL. As a result, multicolor visual emissions can be achieved through co-doping of Tb^3+^ and Eu^3+^ in NaGdF_4_ nanocrystals (Fig. 1A).

**Fig. 1.**
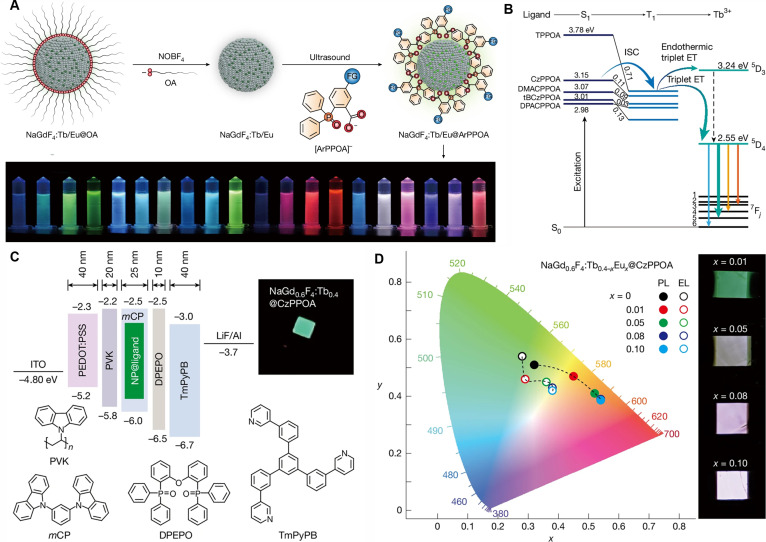
(A) Schematic of the synthetic procedure for NaGdF_4_:Tb^3+^/Eu^3+^ nanocrystals capped with ArPPOA ligands. The bottom panel shows photographs of multicolor emissions from NaGdF_4_:Tb^3+^/Eu^3+^@ArPPOA dispersed in ethanol. (B) Energy level diagram of NaGdF_4_:Tb^3+^@ArPPOA to illustrate the energy-transfer mechanism. (C) Device configuration and energy level diagram for LED devices based on NaGdF_4_:Tb^3+^@ligand as the emissive layer. The chemical structures of the ligands and a photograph of a NaGdF_4_:Tb^3+^@CzPPOA-doped device under operation at 7 V are shown. (D) Variations of CIE1931 chromaticity coordinates for both thin films and devices with increasing Eu^3+^ content. Inset shows the emission photographs of the devices [7]. Copyright 2025, Nature Publishing Group.

Kinetic analysis revealed that coordination of the proposed ligands with Ln^3+^ ions accelerated intersystem crossing (ISC) to the sub-nanosecond range, due to the small singlet–triplet splitting energy and spin-exchange coupling with the 4f electrons of Ln^3+^ ions, which demonstrates that the luminescence of Ln^3+^ ions stems from sensitization by the T_1_ state of the ligand molecules. Additionally, the ISC efficiency from singlet state (S_1_) to T_1_ was determined to be as high as 98.6% in the NaGdF_4_:Tb^3+^@CzPPOA system. Benefiting from the efficient energy transfer from the T_1_ of CzPPOA molecules to the ^5^D_3_ level of Tb^3+^, as revealed by ultrafast spectroscopic measurements, a near-unity triplet transfer efficiency (96.7%) and the highest photoluminescence quantum yield (44.3%) in solution were achieved (Fig. 1B). These results indicate that molecular triplet excitons can be efficiently transferred to Ln^3+^-doped nanoparticles, thus enabling efficient harvesting of luminescence from triplet exciton.

Driven by the remarkable PL properties and improved electroactivity of these nanohybrids, the authors further fabricated 4-layer-structured light-emitting diodes (LEDs) devices, demonstrating efficient LEDs based on NaGdF_4_:Ln^3+^ nanocrystal for the first time (Fig. 1C). The resulting LEDs displayed excellent performance, including a current efficiency (*η*_CE_) of 9.99 cd A^−1^, a power efficiency (*η*_PE_) of 7.66 lm W^−1^, and an external quantum efficiency (*η*_EQE_) of 5.9%. Based on this universal ligand energy-level modulation system, multicolor visible-light emission can be achieved simply by doping different Ln^3+^ ions into NaGdF_4_ nanocrystals, without the need to redesign the device structure (Fig. 1D). This work not only addresses the void in the research area of electroluminescent insulating nanocrystals but also demonstrates the vast potential to break through the intrinsic constraints of materials via rational molecular engineering.

Notably, the critical breakthrough in Ln^3+^ nanocrystal EL was independently and concurrently reported by 2 research groups, underscoring the rapid progress of this field. Yu et al. adopted the same core mechanism based on a LnNP@organic ligand system involving triplet-to-LnNP energy transfer as that in Tan et al.’s work, and focused on narrow-linewidth NIR-II emission (976 to 1,533 nm) via 9-anthracenecarboxylic acid ligands. Tan et al. achieved superior EL efficiency and visible-to-near-infrared multicolor tunability through rationally designed ArPPOA ligands, thus making complementary contributions to the field [8]. However, there remains substantial room for further optimization, such as designing versatile ligands to obtain a lower turn-on voltage, accelerating charge transport to improve electrical conductivity, and optimizing device architecture to integrate with soft matrices. The Ln^3+^-doped fluoride nanocrystal EL system currently faces several inherent challenges. On one hand, the Ln^3+^ ions feature a long radiative lifetime and a low radiative rate, which may impose limitations on luminance and high-speed modulation performance. In the future, the radiative efficiency and light extraction efficiency may be enhanced via the utilization of photonic crystals, micro-lens arrays, and other such structures. On the other hand, device lifetime is a critical prerequisite for commercialization: this research hinges on functionalized ligands, thereby necessitating more robust interfacial chemistry and advanced encapsulation strategies to mitigate the oxidation of organic ligands. Moreover, theoretical studies are essential to elucidate the energy transfer mechanism between organic ligands and Ln^3+^ ions, including the interfacial energy band structure, carrier diffusion length, etc. We anticipate that these efforts will lead to enhanced performance, paving the way for innovative applications in multidisciplinary fields, including micro-LEDs, EL laser devices, and chip-scale circuits for next-generation telecommunications.

## References

[1] Luo J, Li J, Grater L, Guo R, Yusoff AR, Sargent E, Tang J. Vapour-deposited perovskite light-emitting diodes. Nat Rev Mater. 2024;9(4):282–294.

[2] Tao P, Jin J, Zheng X, Pu Y, Wong W. Engineering emissive excited states in organic electroluminescent materials. Matter. 2025;8(6): Article 102142.

[3] Chen D, Chen Y, Zeng G, Zhang D, Lu H. Integration technology of micro-LED for next-generation display. Research. 2023;6: Article 0047. 37223466 10.34133/research.0047 PMC10202190

[4] Gong T, Shi J, Zhang Y, Bai W, Xuan T, Zhou T, Huang K, Xie R. Rare earth-doped perovskite quantum dot microspheres for micro-LED displays. ACS Energy Lett. 2025;10(10):5012–5019.

[5] Zhang H, Zhang H. Special issue: Rare earth luminescent materials. Light Sci Appl. 2022;11(1):260. 36055990 10.1038/s41377-022-00956-9 PMC9440020

[6] Wen G, Abbas N, Xin Y, Wang J, Xiao X, Qi Y, Zhang T, Zhang Z. Lead-free rare-earth based halide double perovskites: From fundamentals, progress to perspectives. Laser Photonics Rev. 2025;19: Article 2500113.

[7] Tan J, Zhang P, Song X, Han C, Wang F, Zhang J, Duan C, Zhang Z, Xu H, Liu X. Electro-generated excitons for tunable lanthanide electroluminescence. Nature. 2025;647(8090):632–638. 41261154 10.1038/s41586-025-09717-1 PMC12629978

[8] Yu Z, Deng Y, Ye J, Turnhout L, Liu T, Tew A, Arul R, Dowland S, Sun Y, Li X, et al. Triplets electrically turn on insulating lanthanide-doped nanoparticles. Nature. 2025;647(8090):625–631. 41261155 10.1038/s41586-025-09601-y PMC12629994

